# Supplementary material for: Learning curve and surgical outcome of robotic assisted colorectal surgery with ERAS program
Source: Sci Rep. 2022 Nov 29;12:20566. doi: 10.1038/s41598-022-24665-w (PMC9709162; doi:10.1038/s41598-022-24665-w)
Supplement: Supplementary file 1 — Supplementary Information 1. [file 41598_2022_24665_MOESM1_ESM.zip › LSC Raw data-Ñ[▒K/102/N_ñw╝╨░O▒Kñσ.pdf]

W52 / W52

臺中榮民總醫院
手術室護理記錄

[redacted]

基本資料

- 室別： 開刀房-06室，手術科別： CRS，手術類別： 常規，日期： 2020/11/05
- 進入時間： 0915 ； 麻醉開始時間： 0920 ； Time Out時間： 1025 ； 劃刀時間： 1026 ； 手術結束時間： 1305 送出時間： 1320 ；
- 麻醉方式： GE
  診斷： Malignant tumor of sigmoid colon
  手術名稱： Laparoscopic Anterior resection and anastomosis - malignant
  手術局部用藥： 無
  術前預防性抗生素：
  - 抗生素名稱一： Veterin 1GM IVD/由麻醉護理師執行
  - 時間： 1015
  - 上次簽章: 陳秋誼

  ，追加預防性抗生素：
- 手術醫師： 蔣鋒帆，林昌霖，林陳彥
- 麻醉醫師： VS 王賜洲
-
  - 刷手護士A ： 邱任宏，時間： 0915，接替刷手一： 林裕瑋，時間： 1200
  - 流動護士A ： 陳秋誼，時間： 0915，接替流動一： 高子貽，時間： 1230

皮膚狀況

A-V Shunt： 無
術前： 完整
記錄者： 上次簽章: 陳秋誼
術後： 完整
記錄者： 高子貽

手術準備

手術臥式 單一手術臥式

-
  - 手術臥式： 截石臥式
  - 使用附件： 溫毯 38 ℃，矽膠枕頭，腳架，其它： 頭架
  - 皮膚準備： 10%Aq Betadine，Hibiscrub，2% Chlorhexidine
  - 電燒： 使用電燒: 電極片位置： 其它： 迴路電擊墊，記錄者： 上次簽章: 陳秋誼

止血帶T： 無

植入物

- 植入物名稱/號碼/數量/部位： Ethicon Endo GIA 60mm-4.1mm x2 , EEA29 x1/ at colon

，記錄者： 上次簽章: 陳秋誼

標本送檢

- 細菌培養 無
- 細胞學 無
- 冰凍切片 無
- 病理標本
  組織名稱一： s colon
  ，
  組織名稱二： distal cut end
  ，
  組織名稱三： IMA Root
  ，更多組織名稱
  - 組織名稱四： pericolic LN
- 截肢 無

標本交付 標本： 4 袋，標本： 0 盒，標本： 0 瓶，細胞學： 0 管，Culture Tube： 0 管，被交付醫師： CR 林陳彥，

- 記錄者： 上次簽章: 高子貽
- 時間： 1250

傷口術後敷料

傷口敷料：

- 請於圖上標示部位：

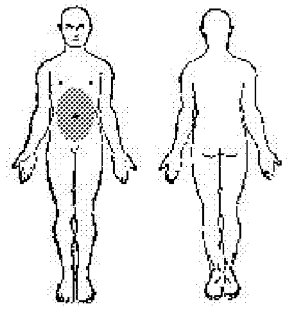

- 傷口敷料： 塗傷口凝膠及貼軟性敷料

導管

導管	天數	導管資訊	插入日期	插入時間	更換日期	拔除日期	拔除時間	拔除原因	預計拔除/更換	預計拔除/更換日期
Epidural PCA(1)	1	部位:脊髓;	2020/11/05	0935						
A-line (1)	1	號碼:20 G; 部位:左手;	2020/11/05	0955						
Double Lumen CVC(1)	1	號碼:8 Fr; 深度:fix15cm; 部位:jugular (右); 象限:RU;	2020/11/05	1000						
Foley(1)	1	號碼:16 Fr.; 部位:膀胱;	2020/11/05	1000						

用物計數

- 手術中出血量： 少 c.c.
- 手術中尿量： 275 c.c.
-
    - 其它： 無

用物計數： 多切口同時縫合

- 1.器械： 正確，2.縫針： 正確，3.刀片： 正確，4.紗布： 正確，5.腹部墊： 未使用，6.紗球： 未使用，7.PEANUTS ： 未使用，8.紗條： 未使用，9.絲帶： 未使用，10.針頭： 未使用，11.Vessel loop： 未使用，12.Boots： 未使用，13.Jelco： 未使用，14.棉條： 未使用，
    - 第一次：
        - 刷手護士 ： 林裕瑋
        - 流動護士： 上次簽章: 陳秋誼
        - 時間： 1200

  ,
    - 第二次：
        - 刷手護士 ： 林裕瑋
        - 流動護士： 上次簽章: 陳秋誼
        - 時間： 1215

  ,
    - 第三次：
        - 刷手護士 ： 林裕瑋
        - 流動護士： 上次簽章: 陳秋誼
        - 時間： 1230

備註

備註： CRS 腹腔鏡 6號

- 記錄者： 上次簽章: 陳秋誼
- 時間： 0940

填寫護理人員 高子貽

正式摘要

		W52 / W52			

doctorRank:B
